# Tributyltin(IV) Butyrate: A Novel Epigenetic Modifier with ER Stress- and Apoptosis-Inducing Properties in Colon Cancer Cells

**DOI:** 10.3390/molecules26165010

**Published:** 2021-08-19

**Authors:** Michela Giuliano, Claudia Pellerito, Adriana Celesia, Tiziana Fiore, Sonia Emanuele

**Affiliations:** 1 Dipartimento di Scienze e Tecnologie Biologiche Chimiche e Farmaceutiche (STEBICEF), Università degli Studi di Palermo, Plesso di Biochimica, Via del Vespro 129, 90127 Palermo, Italy; 2 Dipartimento di Fisica Chimica-Emilio Segrè (DiFC), Università degli Studi di Palermo, Viale delle Scienze, Ed. 17, 90128 Palermo, Italy; claudia.pellerito@unipa.it; 3 CIRCMSB−Consorzio Interuniversitario di Ricerca in Chimica dei Metalli nei Sistemi Biologici, Via Celso Ulpiani, 27, 70125 Bari, Italy; 4 Dipartimento di Biomedicina, Neuroscienze e Diagnostica Avanzata (BIND), Università degli Studi di Palermo, Via del Vespro 129, 90127 Palermo, Italy; adriana.celesia@unipa.it (A.C.); sonia.emanuele@unipa.it (S.E.)

**Keywords:** triorganotin(IV) butyrates, colon cancer, histone acetylation, apoptosis, ER stress, HDAC inhibitors

## Abstract

Organotin(IV) compounds are a class of non-platinum metallo-conjugates exhibiting antitumor activity. The effects of different organotin types has been related to several mechanisms, including their ability to modify acetylation protein status and to promote apoptosis. Here, we focus on triorganotin(IV) complexes of butyric acid, a well-known HDAC inhibitor with antitumor properties. The conjugated compounds were synthesized and characterised by FTIR spectroscopy, multi-nuclear (^1^H, ^13^C and ^119^Sn) NMR, and mass spectrometry (ESI-MS). In the triorganotin(IV) complexes, an anionic monodentate butyrate ligand was observed, which coordinated the tin atom on a tetra-coordinated, monomeric environment similar to ester. FTIR and NMR findings confirm this structure both in solid state and solution. The antitumor efficacy of the triorganotin(IV) butyrates was tested in colon cancer cells and, among them, tributyltin(IV) butyrate (BT2) was selected as the most efficacious. BT2 induced G2/M cell cycle arrest, ER stress, and apoptotic cell death. These effects were obtained using low concentrations of BT2 up to 1 μM, whereas butyric acid alone was completely inefficacious, and the parent compound TBT was poorly effective at the same treatment conditions. To assess whether butyrate in the coordinated form maintains its epigenetic effects, histone acetylation was evaluated and a dramatic decrease in acetyl-H3 and -H4 histones was found. In contrast, butyrate alone stimulated histone acetylation at a higher concentration (5 mM). BT2 was also capable of preventing histone acetylation induced by SAHA, another potent HDAC inhibitor, thus suggesting that it may activate HDACs. These results support a potential use of BT2, a novel epigenetic modulator, in colon cancer treatment.

## 1. Introduction

Organotin(IV) carboxylates possess significant properties such as antibacterial and antifungal agents and display promising antitumor activity [1,2]. Every year, new organotin(IV) carboxylate complexes are synthesized with the aim of improving the anticancer potential of their parental compounds and to find a valid alternative to classical chemotherapeutics that induce serious side effects in patients [3]. In particular, the carboxylic function has been used as an anchor for the tin, leaving the “biological” tasks to other functional groups.

Short-chain fatty acids are naturally occurring fatty acids, and exhibit various pharmacological applications [4,5,6,7]. Since 1860, complexes of organotins with short chain fatty acids have been produced and chemically characterised [8,9].

More recently, Nath has described organotin(IV) complexes with lauric, myristic, and stearic acids through spectroscopic studies [10].

For a long time, we have been involved in organotin(IV) complex synthesis, characterization, and biological studies to test their potential anti-tumor efficacy [11,12,13,14,15,16]. The leitmotiv has been the modulation of the intrinsic organometallic moiety cytotoxicity by means of biologically related molecules (synthetic or natural). Such a modulation is usually achieved by two (often overlapping) routes: by dampening the damaging effects of the metal core and by serving as a carrier for specific tissue districts.

In this work, butyric acid was chosen for its well-known histone deacetylase inhibitory activity, which is strictly correlated with its anti-tumor properties [17].

Butyric acid belongs to short-chain fatty acid class produced by the anaerobic fermentation of dietary fibres in the human colon. Beyond its metabolic function sustaining normal colonocytes, it has been shown to inhibit growth and to induce differentiation of colon cancer cells as well as a variety of other tumor cell types [17]. Butyrate has also been shown to induce apoptosis in tumor cells, an effect which is usually displayed at mM range concentration and which is related with histone hyperacetylation due to histone deacetilase (HDAC) inhibition. However, other mechanisms including butyrate-induced DNA methylation inhibition and post-translational modifications of specific proteins have been reported [18].

Butyrate has also been shown to potentiate the effects of traditional chemotherapeutics such as 5-fluorouracil (5-FU), vincristine, adryamycine, methotrexate, and cisplatin [19,20,21,22]. However, these potentiating effects have also been observed using relatively high concentrations of butyrate.

Considering the low efficacy of butyrate compared to that of the new generation of HDAC inhibitors that have had an impact on epigenetic tumor-targeted therapy [23], we focused on butyrate organotin conjugates to improve its efficacy and to understand whether it maintains its epigenetic properties in colon cancer cells.

Histone modifications provide an important mechanism of epigenetic regulation [24,25] and many organotin compounds may influence epigenetics as well. For instance, evidence has been provided that organotin compounds are capable of affecting histone acetyl transferase (HAT) activity [26] or modifying DNA methylation pattern [27,28].

Our previous studies have clearly shown that organotin complexes with valproic acid, another HDAC inhibitor, exert a remarkable antitumor action in hepatocarcinoma cells, as well as the dibutyltin(IV) complex of caffeic acid [13,15].

Promising results have been obtained with butyrate-releasing prodrugs and butyrate derivatives, rather than with butyrate itself [29,30,31]. In line with these findings, this paper describes triorganotin(IV) butyrates synthesis and its characterization, and provides a biological evaluation of their efficacy in colon cancer cells. Specifically, tributyltin(IV) butyrate was chosen as the most promising compound and its proapoptotic and epigenetic action are described.

## 2. Results and Discussion

### 2.1. Synthesis and Characterization of Triorganotin(IV) Butyrates

The analytical data revealed the formation of compounds with a 1:1 metal-to-ligand ratio, leading to formula R_3_SnBT, R = Me (BT1), Bu (BT2), Ph (BT3), and BT = butyrate.

The structures of the synthesized compounds were confirmed by spectroscopic analyses. The coordinating mode of butyric acid towards the triorganotin(IV) moiety can be inferred by comparing the infrared spectroscopy (IR) spectra of free and coordinated ligands. The vibrational frequencies of OCO, Sn–C, and Sn–O moieties are taken into consideration. These frequencies provide useful information to identify the coordination around tin. In the free ligand, the C=O stretching of the carboxylic group was observed at 1712 cm^−1^. This band disappears upon coordination, indicating deprotonation of the carboxylic group. The characteristic absorption signals of carboxylate group for R_3_Sn butyrate (R = Me, BT1; Bu, BT2; and Ph, BT3) were assigned in the range of 1565–1574 cm^‒1^ for asymmetric stretching and 1338–1345 cm^−1^ for symmetric stretching frequencies. Δ*ν* values (=*ν*_asCOO_^−^ – *ν*_sCOO_^−^) ranging from 220 to 236 cm^−1^ are characteristic for coordinated ester-type carboxylate groups [32,33].

A band in the region of 488–495 cm^−1^ was assigned to *ν*(Sn–O), which supports the bonding of the carboxylate group to the tin atom. The observed *ν*(Sn–C) frequencies in trialkyltin(IV) derivatives (508–545 cm^−1^) and triphenyltin(IV) derivative (at 454 cm^−1^) correspond to the reported values [34,35,36].

**Figure 1:**
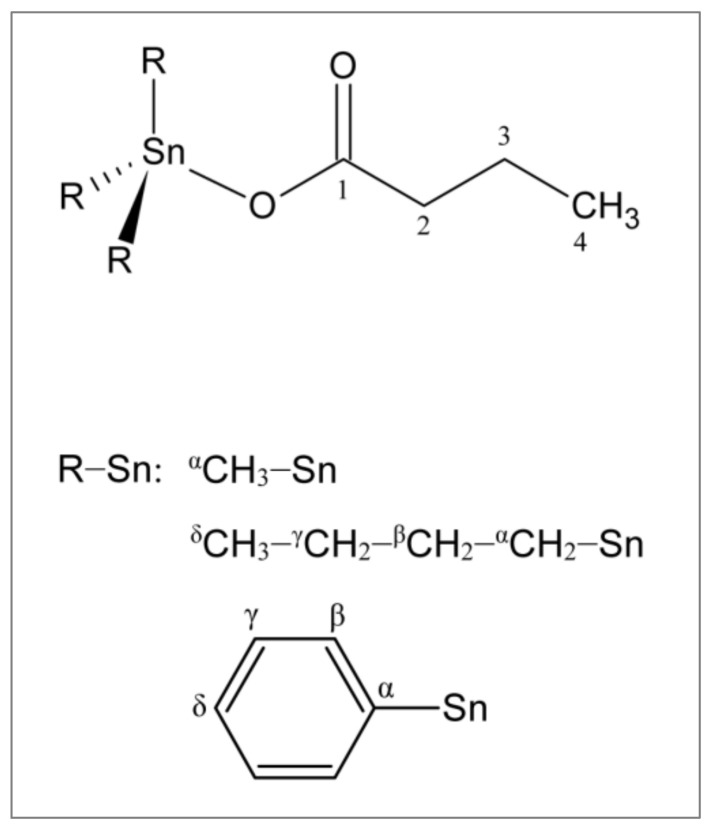
Proposed structure for R_3_Sn(IV) butyrates with the numbering scheme referred to the NMR assignments.

In conclusion, the infrared data suggested a monoanionic monodentate coordination of an unidentate ester-type COO group to the R_3_Sn(IV) moiety tin(IV) atom, resulting in tetra-coordinated, monomeric environment (Figure 1).

The ^1^H and ^13^C {^1^H} NMR spectra of the butyric acid and the studied complexes were recorded in CDCl_3_. Data are given in Materials and Methods (Section 3) and resonances for compounds have been assigned.

The disappearance of the carboxylic proton signal (OH signal at δ 11.85 ppm) for all the spectra of the complexes was diagnostic of the coordination of the ligand upon the triorganotin(IV) moieties. For BT1, the measured ^2^J(^119^Sn, ^1^H) and ^1^J(^119^Sn, ^13^C) satellites observed in the ^1^H and ^13^C spectra, respectively, allowed us to estimate the C–Sn–C angle values of 111° [37,38]. For BT2, only from the ^2^J(^119^Sn,^1^H) satellites, it was possible to calculate a C–Sn–C angle of (113 ± 2)° [39]. These values suggest a local tetrahedral Sn geometry, with butyrate binding the Sn atom conceivably via an ester-like carboxylate. Even in BT3, ^n^J(^119^Sn,^13^C) values (*n* = 1–3), together with the values of the δ(^13^C) values of the α, β, γ, and δ carbon signals, are in agreement with a tetrahedral geometry around the tin center [40].

The ^119^Sn{^1^H} of BT1, BT2, and BT3 showed a single signal at 124.02 ppm, 100.94 ppm and, −117.07 ppm, respectively, and all the values were typical of four coordinated tin centers, in agreement with what was inferred from the estimated C–Sn–C angle values, the δ(^13^C) values, and the ^n^J(^119^Sn,^13^C) values previously discussed.

The electrospray ionization-mass spectrometry (ESI-MS) spectra of BT1, BT2, and BT3 showed very complicated fragmentation patterns because of the presence of several adducts and a wide range of fragment ions in the first-order mass spectra. Nearly all spectra revealed the peaks relative to the monomeric adducts that BT1, BT2, and BT3 form with SnMe_3_, SnBu_3_, and SnPh_3_ groups, respectively, and with alkali metal ions such as Na^+^. Such a complexity is typical of organotin compounds, as previously reported [41,42].

**Figure 2:**
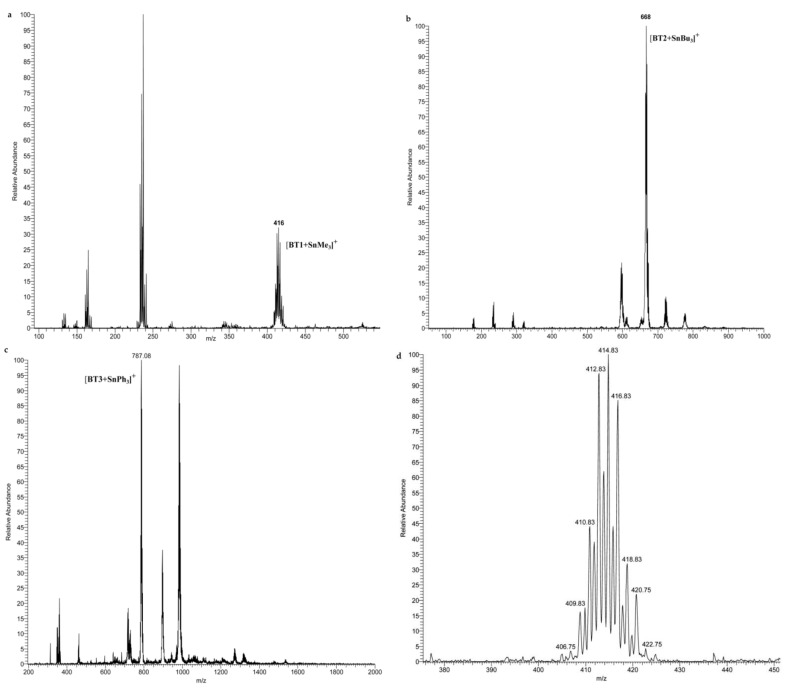
Positive full scan spectra of BT1 (**a**); BT2 (**b**); BT3 (**c**); Compounds and zoom scan spectrum of *m*/*z* 416 [BT1+SnMe_3_]^+^ (**d**).

The assignments of the individual ions were based on the combination of positive-ion, and tandem mass spectrometric experiments, supported by a comparison between theoretical and experimental isotopic distributions of monomeric. Full positive scans of BT1, BT2, and BT3 spectra are reported in Figure 2a–c, and the isotopic distribution of BT1 is showed in Figure 2d.

### 2.2. Biological Study

#### 2.2.1. The Effects of Different Triorganotin(IV) Butyrates on Colon Cancer Cells’ Viability

**Figure 3:**
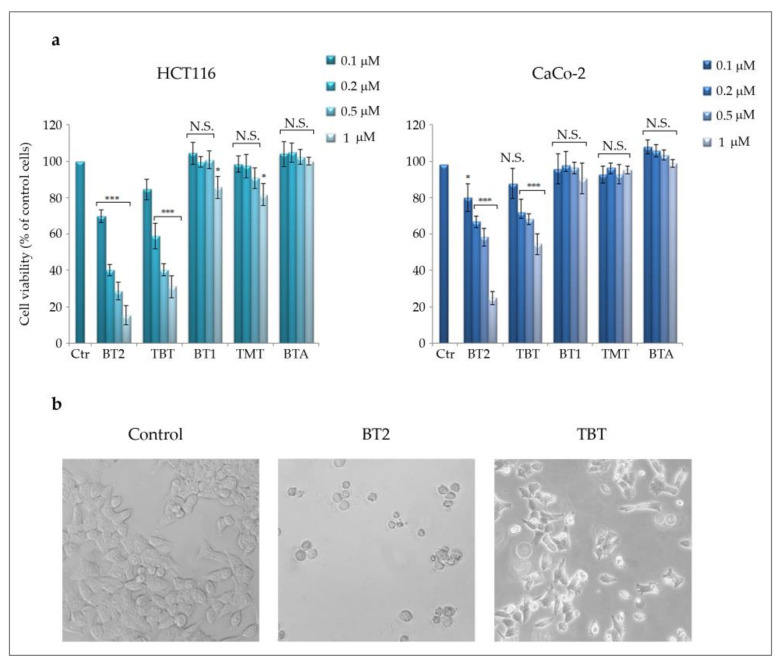
Tributyltin(IV) butyrate (BT2) induces cytotoxic effects in colon cancer cells: (**a**) MTT assay was used to measure cell viability in two colon cancer cell lines (HCT116 and CaCo-2), as reported in Materials and Methods (Section 3). Cells were incubated for 48 h in the presence of the conjugates (BT2 and BT1) and the corresponding parent compounds (TBT and TMT) at the indicated concentrations. The effects of butyric acid (BTA) were also evaluated. The results reported in the histograms are representative of three separate experiments: (*) *p*-value < 0.05; (***) *p*-value < 0.001 compared with untreated cells; N.S. = not significant; (**b**) Morphological analysis of HCT116 cells treated with 0.5 μM BT2 and TBT for 48 h. The cells were visualized under a light microscope at 200× magnification and the pictures were acquired by IM50 Leica Software (Leika Microsystems, Wetzlar, Germany).

As a preliminary screening to test the possible anti-tumor properties of the synthesized compounds, we performed cell viability MTT assay in two colon cancer cell lines, HCT116 and CaCo-2. As Figure 3a shows, tributyltin(IV) butyrate (BT2) presented as the most efficacious among the triorganotin(IV) butyrates analysed, exerting a dose-dependent effect in both cell lines with a maximum effect (−85% in HCT116 and −75% in CaCo-2 cells, respectively) observed with 1 μM after 48 h treatment. The parent compound Bu_3_SnCl (TBT) also reduced cell viability but to a lower extent (−68,9% in HCT116 and −45% in CaCo-2). On the other hand, neither BT1 nor its parent compound Me_3_SnCl (TMT) were significantly active in the same concentration range, being the maximum effect equal to −15% in HCT116 and −8% in CaCo-2 cells for BT1. BT3 was not included in the figure because of its poor solubility in solutions compatible with our experimental conditions. Moreover, the parent compound Ph_3_SnCl (TPT) displayed high toxicity, having determined complete cell death even at 0.1 μM.

It is interesting to note that the butyrate was not capable of determining any effect on cell viability at any concentration used, but data not shown, and in accordance with other observations, indicated that it is active in the range of 1–5 mM in colon cancer cells [43].

Considering these results, we focused the attention on BT2 and we chose HCT116 cells as the most susceptible cell line. Morphological analysis specifically performed in these cells showed that BT2 caused typical features of cell death; cells appeared round shaped and detached from the substrate. Interestingly, the parent compound TBT reduced the cell number, an event that can be considered as in accordance with MTT data, but the cells appeared viable, thus suggesting an anti-proliferative effect (Figure 3b). As for this evaluation, in all subsequent experiments a 0.5 μM concentration was chosen to compare the effects of BT2 and TBT.

#### 2.2.2. The Effect of BT2 on Cell Cycle and ER Stress

**Figure 4:**
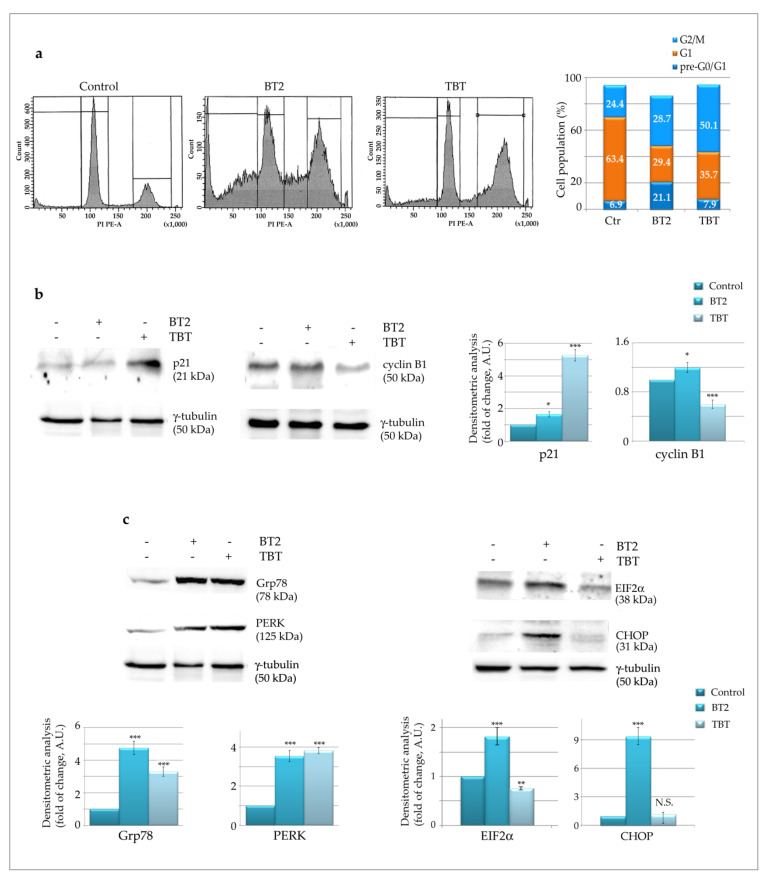
Tributyltin(IV) butyrate (BT2) induces cell cycle arrest accompanied by ER stress induction and DNA fragmentation: (**a**) The cell cycle phase distribution of HCT116 cells was evaluated by flow cytometry analysis. Cells were incubated for 48 h in the presence of 0.5 μM BT2 or TBT. Then, the DNA content was evaluated after incubating the cells in a hypotonic propidium iodide solution, as described in Section 3. Fluorescence was estimated by FacsDiva Software. (**b**,**c**) Western blot analysis of p21 and cyclin B1, two proteins differently expressed during the cell cycle phases (**b**), and Grp78, PERK, Phospho eIF2α, and CHOP markers of endoplasmic reticulum stress (**c**). Cells were incubated for 48 h in the presence of 0.5 μM BT2 or TBT. The correct protein loading was ascertained by evaluating γ-tubulin levels. Representative blots of three independent experiments and densitometric analysis are shown. (*) *p*-value < 0.05; (**) *p*-value < 0.01; (***) *p*-value < 0.001 compared with untreated cells. N.S. = not significant.

To investigate the difference between the effects of BT2 and its parent TBT, we analysed the cell cycle distribution profiles. Data reported in Figure 4a shows that BT2 induced a marked increase in the pre-G0/G1 peak (21.1% compared to 6.9% in the control), indicating DNA fragmentation that can be associated with cell death. In addition, a slight increase in the G2/M phase was observed in this condition (from 24.4% in the control to 28.7 with BT2), suggesting a possible shift to cell death following cell cycle arrest in this phase. Concerning the parental compound TBT, G2/M phase arrest was predominant (50.1%), whereas DNA pre-G0/G1 was quite modest (7.9%). Such a result is in line with the anti-proliferative effect of TBT, which was not accompanied with cell death. Biochemically, it is interesting to observe that only TBT induced a marked increase in the cyclin-dependent kinase (CDK) inhibitor p21 and a concomitant decrease in the levels of cyclin B1 (Figure 4b). Both sets of data nicely correlate with G2/M arrest, as previously described by Lallemand et al. [44]. In contrast, BT2 only slightly modified p21 and cyclin B1 levels, a result that can be interpreted considering the slight increase in G2/M phase and the predominance of cell death induced by the compound.

Evidence has been provided that G2/M arrest can correlate with ER stress and possibly with subsequent apoptosis [45]. Therefore, we analysed some important ER stress markers, such as the 78 kDa glucose-regulated protein (Grp78), the initiator of the unfolded protein response (UPR), RNA-dependent protein kinase (PKR)-like ER kinase (PERK), its target eukaryotic initiation factor 2 (eIF2*α*) in the phosphorylated form, and the downstream transcription factor C/EBP homologous protein (CHOP). The levels of Grp78 and PERK markedly increased following treatment with BT2 or TBT (Figure 4c), indicating that ER stress occurs in both conditions, most likely correlating with the organotin moiety. However, increase in the levels of phosphorylated IF2*α* and CHOP was only observed following the BT2 treatment. It is thus possible to deduce that UPR was effectively triggered following BT2 treatment and not TBT, an observation which is in line with the ability of BT2 to promote cell death. This conclusion is also supported by the evidence that CHOP represents a key transcription factor that correlates persistent ER stress with the induction of apoptosis.

In order to characterise BT2-induced cell death and verify apoptosis induction, we made further biochemical evaluations.

#### 2.2.3. BT2 Promotes Apoptotic Cell Death

**Figure 5:**
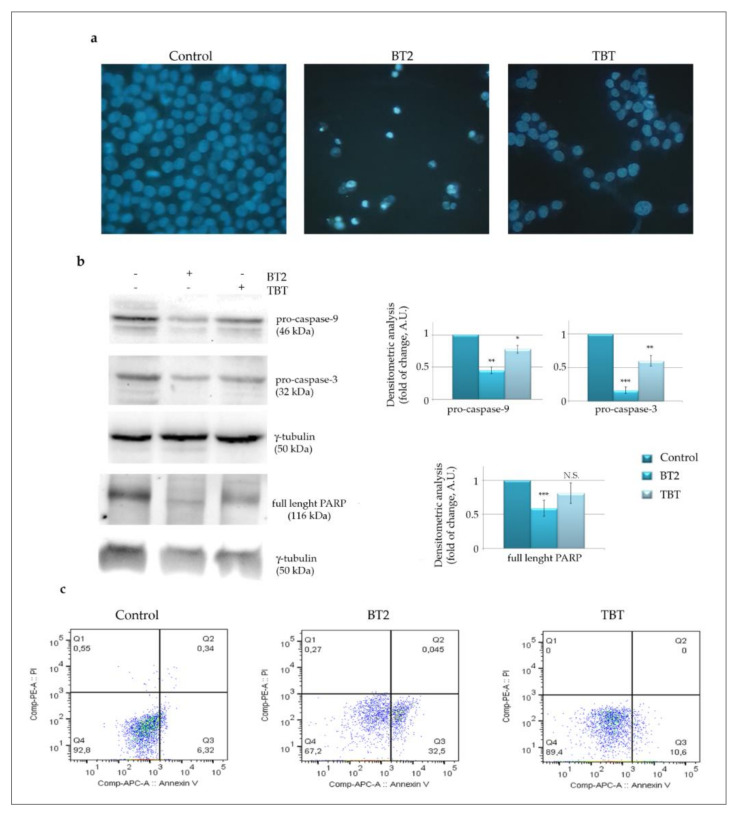
Tributyltin(IV) butyrate (BT2) activates caspase-dependent apoptotic cell death pathway. (**a**) Cells were incubated for 48 h in the presence of 0.5 μM BT2 or TBT. At the end of incubation, cells were stained with the vital dye Hoechst 333428 that permits to visualize nuclei. Cells were then visualised under fluorescence microscope Leika equipped with a DAPI filter (magnification of ×400). Micrographs are representative of almost two fields from two independent experiments; (**b**) Western blot analysis of apoptotic markers, pro-caspase-9, pro-caspase-3 and PARP, in cells treated as in (**a**). The correct protein loading was ascertained by evaluating γ-tubulin levels. Representative blots of three independent experiments and densitometric analysis are shown; (**c**) AnnexinV positivity confirmed early apoptosis. Cells were treated with the compounds for 24 h and subjected to Annexin V apoptosis detection kit as reported in Materials and methods. Analysis was performed by flow cytometry using FacsCanto BD. The percentage of annexin V positive cells was evaluated by Flowjo BD software. The results are representative of two independent experiments (*) *p*-value < 0.05; (**) *p*-value < 0.01; (***) *p*-value < 0.001 compared with untreated cells. N.S. not significant.

In order to confirm the morphological data suggesting that BT2 induces cell death, we stained the cells with Hoechst, a vital dye that highlights the nuclei. As shown in Figure 5a, BT2 treatment produced a marked effect of chromatin condensation and fragmentation, evidenced as bright spots, whereas TBT only reduced the cell number compared to the control, with the nuclei appearing still intact. Moreover, investigating apoptotic markers by Western blot analysis revealed that BT2 was capable of reducing the levels of pro-caspase-9 and pro-caspase-3, an indication of their activation (Figure 5b). These effects were less evident with TBT. We also focused on poly-ADP ribose polymerase (PARP), a well-known caspase-3 substrate. A significant reduction in PARP level was observed following BT2 treatment and this effect was not obtained with TBT. However, in this condition PARP cleavage fragments were not detected, suggesting that complete PARP degradation possibly occurred. We cannot exclude the intervention of other proteases in PARP degradation, in accordance with the observations of Chaitanya et al. [46].

To further confirm apoptosis induction, we stained the cells with annexinV, an early apoptotic indicator that detects the exposure of phosphatidylserine to the outer side of the plasma membrane, an event which is associated with classic apoptosis commitment. 

The results shown in Figure 5c indicate that only BT2 significantly increased the percentage of annexin V positive cells at 24 h treatment (32.5% compared to the control 6.32%).

Interestingly, the parental compound TBT did not significantly increase annexin V positivity (only 10.6%), thus confirming that apoptosis is specifically triggered by BT2 only.

#### 2.2.4. BT2 Induces Histone Deacetylation

It is well known that butyrate behaves as an epigenetic compound since at mM concentration range it is capable of inhibiting HDACs with consequent histone hyper-acetylation [17,47]. Moreover, evidence has been provided that butyrate, at low concentrations, activates histone acetyl transferases (HATs), being metabolized to acetyl-CoA, which is important not only as an energy source but also as HATs substrate [47].

In order to investigate whether BT2 also exerts an epigenetic effect, we first evaluated histone acetylation pattern in comparison with butyric acid. To this purpose, we analysed acetyl-H3 and acetyl-H4 histones, two major substrates of both HATs and HDACs.

**Figure 6:**
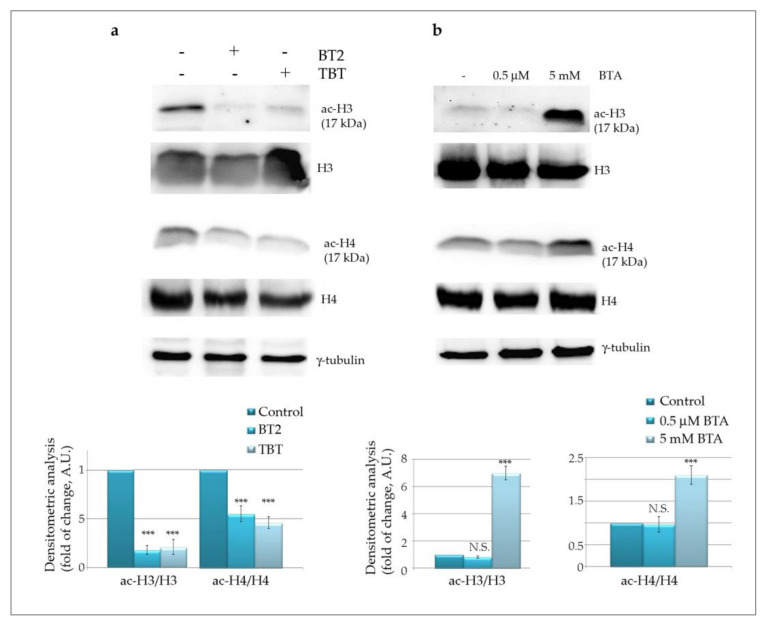
Tributyltin(IV) butyrate (BT2) remarkably reduces histone acetylation. Western blot analysis of acetylated-H3 and H4 histones after treatment for 48 h with 0.5 μM BT2 and TBT (**a**) or 0.5 μM and 5 mM butyric acid (BTA) (**b**) are shown. The ratio between acetylated histones and total histone levels was quantified. Representative blots of three independent experiments and densitometric analysis are shown. (***) *p*-value < 0.001 compared with untreated cells. N.S. = not significant.

Surprisingly, the results shown in Figure 6a clearly indicate that BT2 dramatically reduces histone acetylation, an effect that was also observed with the parental compound TBT. On the other hand, butyric acid, at the same concentration (0.5 μM), was not capable of exerting any significant effect whereas at 5 mM produced the typical remarkable increase in both acetyl-H3 and acetyl-H4 histones (Figure 6b). It is noteworthy that the level of total H3 and H4 histones, considered as loading controls, was just slightly reduced by the treatment with the compounds. Densitometric analysis performed considering the ratio between acetylated/total histones confirmed that the acetylated forms markedly decreased.

Since histone deacetylation was obtained with both BT2 and TBT, these data may be interpreted as a specific effect of the tributyltin moiety. If this hypothesis is correct, it is possible to assume that the BT2 compound does not maintain the deacetylation inhibiting activity of its ligand butyrate.

In order to verify whether BT2 produces histone deacetylation by activating HDACs, we performed experiments in the presence of suberoylanilide hydroxamic acid (SAHA), another potent HDAC inhibitor [48].

**Figure 7:**
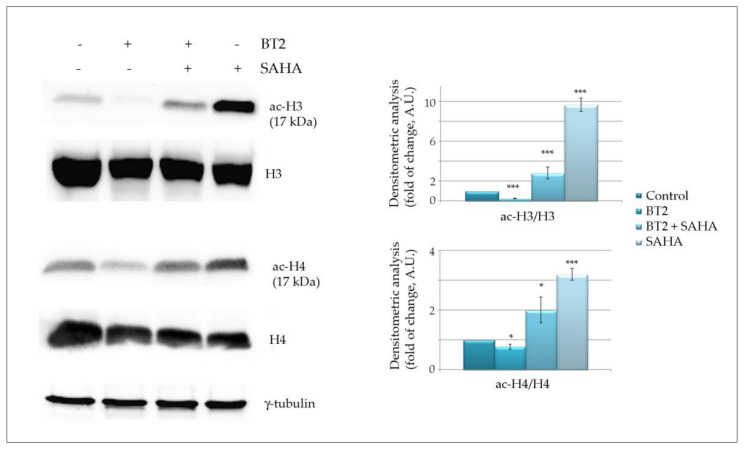
Tributyltin(IV) butyrate (BT2) reduces the effect of the HDAC inhibitor SAHA on histone acetylation. The figure shows Western blot analysis of acetylated-H3 and H4 histones after cell treatment for 48 h with 0.5 μM BT2 (lane 2) or 24 h treatment with 10 μM SAHA used alone (lane 4) or after pre-treatment with BT2 (24 h) followed by other 24 h of incubation with the HDAC inhibitor (lane 3). The ratio between acetylated histones and total histone levels was quantified. Representative blots of three independent experiments and densitometric analysis are shown. (*) *p*-value < 0.05; (***) *p*-value < 0.001 compared with untreated cells. N.S. = not significant.

To see whether BT2 could specifically prevent SAHA-induced histone acetylation, we pre-treated the cells for 24 h with BT2 and added SAHA for the successive 24 h. Data reported in Figure 7 confirms that BT2 alone markedly reduces the levels of both acetyl H3 and acetyl H4. Conversely, SAHA alone produced a remarkable increase in histone acetylation. Interestingly, pre-treatment with BT2 was capable of counteracting SAHA-mediated deacetylation of both histones. Considering the well-known HDAC inhibitory action of SAHA, these data strongly suggest that BT2 may act as an HDAC activator.

Further study specifically focusing on epigenetic profile of BT2 action will verify this hypothesis and better characterise the effects of the tributyltin moiety.

## 3. Materials and Methods

### 3.1. Materials

Reagents Me_3_SnOH, (Bu_3_Sn)_2_O, Ph_3_SnOH, and BTA used in the experiment were purchased at commercial analytical grade from commercial sources (Sigma–Aldrich, St. Louis, Missouri, USA) and used directly without further purification.

### 3.2. Synthetic Procedures of Triorganotin(IV) Complexes

Triorganotin(IV) complexes of butyric acid (BTA) were obtained by the same method: BTA (0.356 g, 4 mmol) was added to a methanol solution (200 mL) with the stoichiometric amount for the corresponding triorganotin oxide or hydroxide (Me_3_SnOH: 0.7228 g, 4 mmol; (Bu_3_Sn)_2_O: 1.2420 g, 2 mmol; Ph_3_SnOH: 1.4681 g, 4 mmol, respectively, for compounds BT1, BT2, and BT3). The mixtures were kept refluxing overnight. White solids were recovered upon cooling after concentration in a rotary evaporator. After filtration and vacuum drying, the solids were recrystallized from petroleum ether and analyzed.

#### 3.2.1. Trimethyltin(IV) butyrate (BT1)

Physical state: white solid; yield: 82%. m.p. 124–126 °C. Formula Weight, FW = 250.91. Anal. Calc. for C_7_H_16_O_2_Sn: C 33.51; H 6.43; Sn 47.31%; Found: C 33.74; H 6.47; Sn 47.27%. IR (KBr, cm^−1^): 1565 ν_as_ (COO^−^), 1345 ν_s_ (COO^−^), 545 (Sn–C), 488 (Sn–O), 220 Δν. ^1^H NMR (CDCl_3_, ppm): 2.24 (t, 2H, CH_2_(2) ligand); 1.60 (m, 2H, CH_2_(3) ligand); 0.90 (t, 3H, CH_3_(4) ligand); 0.51 (s, 9H, CH_3_-α; ^2^J(^119^Sn–^1^H) = 58 Hz). ^13^C NMR (CDCl_3_, ppm): 181.23 (COOH); 39.57 (C2 ligand); 21.53 (C3 ligand); 16.58 (C4 ligand); 0.09 (CH_3_-α, ^1^J(^119^Sn–^13^C), 400 Hz). ^119^Sn NMR (ppm): 124.02. ESI-MS: *m*/*z* 416 [BT1+SnMe_3_]^+^; *m*/*z* 274 [BT1+Na]^+^; *m*/*z* 236 [BT1-CH_3_]^+^; *m*/*z* 165 [SnMe_3_]^+^. MS/MS of *m*/*z* 237: *m*/*z* 206 [BT1-3CH_3_]^+^; *m*/*z* 165 [SnMe_3_]^+^; *m*/*z* 150 [SnMe_2_]^+^. MS/MS of *m*/*z* 237: *m*/*z* 150 [SnMe_2_]^+^.

#### 3.2.2. Tributyltin(IV) butyrate (BT2)

Physical state: white solid; yield: 85%. m.p. 68–69 °C. Formula Weight, FW = 377.15. Anal. Calc. for C_16_H_34_O_2_Sn: C 50.95; H 9.09; Sn 31.48%; Found: C 51.21; H 9.12; Sn 31.27%. IR (KBr, cm^−1^): 1574 ν_as_ (COO^−^), 1338 ν_s_ (COO^−^), 508 (Sn–C), 488 (Sn–O), 236 Δν. ^1^H NMR (CDCl_3_, ppm): 2.26 (t, 2H, CH_2_(2) ligand); 1.60 (m, 8H, 2 × CH_2_(3) ligand and 6 × CH_2_-α; ^2^J(^119^Sn–^1^H) = 70 Hz); 1.31 (m, 6H, CH_2_-β); 1.23 (m, 6H, CH_2_-γ); 0.89 (t, 12H, 3 × CH_3_(4) ligand and 9 CH_3_-δ). ^13^C NMR (CDCl_3_, ppm): 181.85 (COOH); 39.28 (C2 ligand); 30.43 (C-β); 29.57 (C-γ); 21.84 (C3 ligand); 18.99 (C-α); 16.26 (C4 ligand); C-δ not observed. ^119^Sn NMR (ppm): 100.94. ESI-MS: *m*/*z* 777 [2BT2+Na]^+^; *m*/*z* 720 [2BT2-butyl+Na]^+^; m/z668 [BT2+SnBu_3_]^+^; *m*/*z* 600 [2BT2-SnBu+Na]^+^; *m*/*z* 320 [BT2-butyl]^+^; *m*/*z* 291 [SnBu_3_]^+^; *m*/*z* 234 [SnBu_2_]^+^; *m*/*z* 177 [SnBu]^+^. MS/MS of *m*/*z* 777: *m*/*z* 400 [BT2+Na]^+^; *m*/*z* 291 [SnBu_3_]^+^. MS/MS of *m*/*z* 720: *m*/*z* 378 [BT2+H]^+^; *m*/*z* 320 [BT2-butyl]^+^. MS/MS of *m*/*z* 668: *m*/*z* 320 [BT2-butyl]^+^; *m*/*z* 291 [SnBu_3_]^+^; *m*/*z* 234 [SnBu_2_]^+^. MS/MS of *m*/*z* 600: *m*/*z* 291 [SnBu_3_]^+^; *m*/*z* 234 [SnBu_2_]^+^.

#### 3.2.3. Triphenyltin(IV) butyrate (BT3)

Physical state: white solid; yield: 84%. Decomposition temperature: 218–220 °C. Formula Weight, FW: 437.12. Anal. Calc. for C_22_H_22_O_2_Sn: C 60.45; H 5.07; Sn 27.16%; Found: C 60.28; H 4.96; Sn 27.43%. IR (KBr, cm^−1^): 1573 ν_as_ (COO^−^), 1342 ν_s_ (COO^−^), 495 (Sn–O), 454 (Whiffen Y–mode [36]), 231 Δν. ^1^H NMR (CDCl_3_, ppm): 7.73 (m, 6H, H-β aromatic, ^3^J(^119^Sn-^1^H), 54.0 Hz); 7.44 (m, 9H, 6 × H-γ, 3 × H-δ aromatic); 2.40 (t, 2H, CH_2_(2) ligand); 1.68 (m, 2H, CH_2_(3) ligand); 0.92 (t, 3H, CH_3_(4) ligand). ^13^C NMR (CDCl_3_, ppm): 181.54 (COOH); 141.06 (C-α aromatic, ^1^J(^119^Sn–^13^C) = 652.4 Hz); 139.79 (C-β aromatic, ^2^J(^119^Sn–^13^C) = 47.5 Hz); 132.66 (C-δ aromatic); 131.46 (C-γ aromatic, ^3^J(^119^Sn–^13^C) = 64.4 Hz); 38.62 (C2 ligand); 21.80 (C3 ligand); 16.39 (C4 ligand). ^119^Sn NMR (ppm): −117.07. ESI-MS: *m*/*z* 983 [BT3+SnPh_3_+SnPh]^+^; *m*/*z* 897 [2BT3+Na]^+^; *m*/*z* 788 [BT3+SnPh_3_]^+^; *m*/*z* 719 [(SnPh_3_)_2_O+H]^+^; *m*/*z* 460 [BT3+Na]^+^; *m*/*z* 360 [BT3-benzene]^+^; *m*/*z* 351 [SnPh_3_]^+^. MS/MS of *m*/*z* 788: *m*/*z* 360 [BT3-benzene]^+^; MS/MS of *m*/*z* 719: *m*/*z* 642 [(SnPh_3_)_2_O-benzene]^+^; *m*/*z* 351 [SnPh_3_]^+^. MS/MS of *m*/*z* 351: *m*/*z* 195 [SnPh]^+^.

### 3.3. Instrumentation

All melting points were measured on a Stuart SMP3 melting point apparatus Bibby Scientific Ltd., Stone, UK.

The IR spectra were recorded in wavenumber (ν, cm^−1^) as nujol and hexachlorobutadiene mulls on a Frontier Spectrophotometer PerkinElmer FTIR (Milan, Italy) between CsI windows in the 4000–250 cm^−1^ region and in potassium bromide (KBr) disks in the range 4000−400 cm^−1^. 

^1^H, ^13^C{^1^H} NMR spectra were recorded on a Bruker ARX 300 (7.04 T) spectrometer (Rheinstetten, Germany), at 300 K. One-dimensional ^1^H, ^13^C{^1^H} spectra in deuterated chloroform (CDCl_3_) solution were acquired at 400.15 and 100.61 MHz, with a SW of 10 ppm and 200 ppm for ^1^H and ^13^C{^1^H}, respectively. ^1^H and ^13^C resonances were calibrated on the corresponding solvent signals, ^1^H, δ = 7.27 ppm; ^13^C, δ = 77.32 ppm with respect to the Me_4_Si (TMS) [49].

Solution ^119^Sn{^1^H} NMR data were recorded on Bruker Avance II 400 MHz (9.40 T) spectrometer (Bruker BioSpin GmbH, Rheinstetten, Germany) at 300 K, in CDCl_3_. One-dimensional ^119^Sn{^1^H} spectra were acquired at 149.21 MHz with a spectral width (SW) of 800 ppm by investigating four spectral windows with SW = 250 ppm at once in the +200:−600 ppm range. For ^119^Sn, Me_4_Sn was used as external reference (^119^Sn δ = 0.00 ppm).

^119^Sn{^1^H} and ^13^C{^1^H} spectra were acquired with broadband proton power-gated decoupling. For all nuclei, positive chemical shift had higher frequencies than the reference. The coupling constants (J) were expressed in Hz; the splitting patterns were designated as s (singlet), t (triplet), and m (multiplet). All the samples were ca. 0.05 M.

ESI-MS spectra were recorded on a Finnigan LCQ Deca XP ion trap (Thermo Fisher Scientific, Waltham, Massachusetts USA) using an electrospray ionization (ESI) interface. Complexes were dissolved in methanol or acetonitrile and introduced into the ESI source via a 100 μmi.d. fused silica capillary using a 500 μL syringe. The experimental conditions for spectra, acquired in positive ion mode, were as follows: needle voltage 3.5 kV; flow rate 3–5 μL min^−1^; source temperature 220 °C; *m*/*z* range 50:2000; cone potential 43 V; tube lens offset 55 V.

Elemental analyses for C and H content were performed at Laboratorio di Chimica Organica, Università di Padova. The tin content was determined in our laboratory, gravimetrically as SnO_2_, according to Neumann’s method [50].

### 3.4. Cell Cultures

Human colon cancer HCT116 and CaCo-2 were cultured in RPMI 1640 medium containing 10% (*v*/*v*) fetal bovine serum (FBS), 2.0 mM glutamine and antibiotic, and antimycotic solution (100 U/mL penicillin, 100 μg/mL streptomycin) in a humidified atmosphere at 37 °C in the presence of 5% CO_2_. For the experiments, cells were seeded at 60–70% confluence in 96- or 6-well-plates. Cells were then allowed to adhere overnight and subsequently treated with the compounds or vehicle alone. For in vitro experiments, triorganotin(IV) butyrates were dissolved in DMSO and prepared as 10 mM stock solutions, which were opportunely diluted in culture medium prior to use. The final concentration of DMSO in the incubation mixture did not exceed 0.04% (*v*/*v*), which was verified not to affect cellular functions. As a control, equal volumes of DMSO were added to untreated cells.

### 3.5. Cell Viability MTT Assay Evaluation

Cell viability was evaluated by 3-(4,5-dimethylthiazol-2-yl)-2,5-diphenyltetrazolium bromide (MTT) assay, as previously described [16]. Briefly, after treatment in 96-well plates (triplicate wells for each sample), MTT solution (final concentration 1 mg/mL) was added for 2 h. The medium was then replaced with lysis buffer (100 μL) and the absorbance at 570 nm (test wavelength) and at 630 nm (reference wavelength) was measured using an enzyme-linked immune sorbent assay (ELISA) microplate reader (Dynex Technologies, Chantilly, VA, USA). Values reported in the figures are expressed as percentage of the viability of untreated cells and are the means ± SD of four independent experiments.

### 3.6. Cell Cycle Evaluation

Cell cycle distribution was measured after cell trypsinization, (0.025% trypsin-EDTA) cell harvest, washings in PBS, and the addition of a hypotonic solution containing 25 μg/mL propidium iodide, 0.1% sodium citrate, 0.01% Nonidet P-40, and 10 μg/mL RNase A. The cell cycle phase distribution was evaluated by FacsCanto cytometer (Becton Dickinson, Milan, Italy) using FacsDiva Software (Becton Dickinson, Milan, Italy). Cell debris and aggregates were excluded by opportune gating and 5000 events for each sample were analysed. The results shown in the figure are representative of three independent experiments.

### 3.7. Evaluation of Cell Death

Cell death was assessed by staining the cells with the vital dye Hoechst 33342, which evidences nuclei and allows for the detecting of chromatin condensation and fragmentation. For these experiments, 7 × 10^3^ cells/well were seeded in a 96-well plate, incubated with the compounds for the established times and then stained with Hoechst (2.5 µg/mL medium) for 30 min. After washings with PBS, cells were visualised using an inverted Leica fluorescent microscope (Leica Microsystems, Wetzlar, Germany) endowed with a 4’,6-diamidino-2-phenylindole dihydrochloride (DAPI) filter. Leica Q Fluoro Software (Leica Microsystems, Wetzlar, Germany) was used for image acquisition. Annexin V apoptosis detection assay was used to evidence early apoptotic cells. Briefly, 3 × 10^5^ cells were seeded in 6 cm diameter Petri dishes, allowed to adhere overnight and then treated with the compounds for 24 h. Cells were then harvested, washed twice in PBS, and 10^5^ were incubated for 15 min with 5 µL annexinV/PI in a 100 µL binding buffer. Following dilution (500 µL binding buffer final volume) analysis was performed by flow cytometry using FacsCanto BD. The percentage of annexin V positive cells was evaluated by Flowjo BD Software, Milan, Italy.

### 3.8. Western Blotting Analysis

After treatment, protein extracts were prepared by incubating the cells for 20 min in an ice-cold lysis buffer supplemented with a protease inhibitor cocktail, as previously reported [51]. After sonication (10 s, three times) and protein quantification by Bradford assay, an equal amount of proteins (40 μg) was separated by sodium dodecyl sulphate-Polyacrylamide gel electrophoresis (SDS-PAGE) and then electrotransferred to a nitrocellulose membrane for immunodetection. The blots were developed using electrochemical luminescence labeling systems by ChemiDoc, XRS (Bio-Rad, Hercules, CA, USA) Image system. Optical densities of the bands were analysed with Quantity One Imaging Software (Bio-Rad Laboratories). Both Ponceau red staining and immunoblotting for the housekeeping protein γ-tubulin were considered to verify the correct protein loading. The results shown in the figures are representative of three independent experiments with similar results. Densitometric analysis are reported as the ratio of the intensity of the bands of treated samples to untreated ones after normalization with γ-tubulin. For acetylated histones, normalization was made with total histone levels.

### 3.9. Statistical Analysis

Data were represented as mean ±S.D., and analysis was performed using the Student’s *t*-test and one-way analysis of variance. Comparisons between the control (untreated) vs. all treated samples were made. If a significant difference was detected by ANOVA analyses, this was re-evaluated by post-hoc Bonferroni’s test. GraphPadPrismTM 4.0 Software (Graph PadPrismTM Software Inc., San Diego, CA, USA) was used for statistical calculations. The statistical significance threshold was fixed at *p* < 0.05.

## 4. Conclusions

Overall, our results indicate for the first time that tributyltin(IV) butyrate exerts anti-tumor properties in colon cancer cells. This action is correlated with the G2/M arrest of the cell cycle with consequent ER stress and apoptosis. The compound also exerted an epigenetic effect consisting in histone deacetylation, in contrast with butyric acid that instead determined histone acetylation due to HDAC inhibition. Although different mechanisms, such as histone acetyl transferase (HAT) inhibition or a reduced acetyl CoA availability, can account for histone deacetylation, a suggestive hypothesis consists in an opposite behaviour that the butyrate derivative BT2 acquires compared to the parental butyrate, most likely due to HDAC activation (rather than inhibition) by the compound in the conjugated form.

## Data Availability

Not applicable.

## Sample Availability

Samples of triorganotin(IV) butyrates are available from the authors.
